# Neonatal ten-year retrospective study on neural tube defects in a second level University Hospital

**DOI:** 10.1186/s13052-020-00836-1

**Published:** 2020-05-24

**Authors:** Ettore Piro, Gregorio Serra, Ingrid Anne Mandy Schierz, Mario Giuffrè, Giovanni Corsello

**Affiliations:** Department of Health Promotion Sciences, Maternal and Infant Care, Internal Medicine and Medical Specialties “G. D’Alessandro”, University of Palermo, Piazza delle Cliniche, 2, 90127 Palermo, Italy

**Keywords:** NTDs, Spina bifida, Newborn, Clinical management, Neurodevelopmental follow-up, Prevention

## Abstract

**Background:**

Aim of this retrospective study was to describe clinical characteristics, diagnostic work-up, management and follow-up of newborns with neural tube defects (NTDs), admitted to the Mother and Child Department of the University Hospital of Palermo, in a ten years period.

**Methods:**

The medical records of 7 newborns (5 males and 2 females) admitted, over a 10-year period from January 2010 to March 2020, to our Department on the first day of life were reviewed. Analyzed data were related to familiar and/or maternal risk factors (consanguinity, maternal preexisting and/or gestational diseases, exposure to teratogen/infectious agents, lack of preconception folic acid supplement), demographic (ethnicity/origin, residence) and clinical features (eventual use of assisted reproduction techniques, prenatal diagnosis, gestational age, fetal presentation, type of delivery, birth weight, preoperative imaging, antibiotics and analgesics use, description of the surgery intervention, length of hospital stay, comorbidities, complications), and follow-up.

**Results:**

In our sample we observed a wide spectrum of NTDs: 3 newborns had open NTDs, namely myelomeningocele (2 lumbosacral, one of which associated with extradural lipoma, and 1 sacral), and 4 closed ones, including 2 with meningocele (occipital), 1 filar lipoma associated with dermal sinus, and 1 terminal myelocystocele. Our patients were discharged between 8 and 22 days of life.

The neurodevelopmental follow-up showed a favorable outcome for 4 of the 7 patients, and the appearance over time of neurological impairment (motor and/or autonomic) in the newborns with open NTDs.

**Conclusions:**

This study describes familiar and/or maternal risk factors and demographic and clinical features of a single-center series of newborns with NTDs. It may provide a further outline of the actual phenotypic spectrum of these malformations, and new insights into epidemiological aspects and comprehensive management of the patients, including diagnostic work-up and follow-up evaluations.

## Background

Neural tube defects (NTDs) are congenital malformations of the central nervous system [1]. They are caused by partial/incomplete closure of the neural tube during embryogenesis, between 21 and 28 days after conception [2, 3]. Disorders of primary neurulation include craniorachischisis in which the neural tube fails to initiate closure, leaving most of the brain and the entire spine open. If closure initiates successfully, then the cranial and/or spinal neural folds may fail to close generating exencephaly/anencephaly and open spina bifida (myelomeningocele). Malformations resulting from disturbance of secondary neurulation are closed (skin covered) and often involve tethering of the spinal cord, with associated ectopic lipomatous material [4, 5]. Nevertheless, recent evidences support a post-neurulation origin for encephalocele and demonstrate that brain herniation and failure of brain/spine neural tube closure can all occur as possible developmental outcomes of an identical genetic defect [6]. Infants with anencephaly are mostly stillborn, or die shortly after birth, while those with spina bifida and encephalocele may survive, although suffering from physical and developmental disabilities of various degrees of severity [7]. Worldwide average incidence is 1.8/1000 births (more than 300,000 affected infants born every year), with significant geographic (ethnic, environmental, socioeconomic) variations (> 1 to around 10/1000 births in middle and low-income countries (LMICs) and decreasing incidence in high-income countries (HICs, 0.6/1000 in USA) in the recent decades [8, 9].

NTDs are among the main causes of childhood mortality and disability worldwide, with higher rates in LMICs for limited access to specialized neonatological/pediatric and surgical care [8–10]. Moreover, inequities in the access to high quality care affect pregnant/childbearing age women [11], in relation to low rate of prenatal diagnosis and prevention tools (preconception folic acid supplement), thus explaining the higher prevalence of NTDs at birth.

Here we report on a ten years retrospective study of newborns with NTDs assisted at the Mother and Child Department of the University Hospital of Palermo (Sicily, Italy). The aim of the study was to describe epidemiological aspects and clinical characteristics of these patients, as well as diagnostic work-up, comprehensive management and updated follow-up. To the best of our knowledge, our report is the first in Western Sicily concerning these malformations.

## Methods

### Patients

This is a retrospective study, conducted over a 10-year period from January 2010 to March 2020, on all newborns with neural tube defects (NTDs) admitted to the Mother and Child Department of the University Hospital of Palermo (Sicily, Italy). Our Department includes 3 Operative Units: Obstetrics and Gynecology, Neonatology and Neonatal Intensive Care and Pediatric Surgery. The latter is one of the few in Sicily (which is among the most populated Italian regions, with around 5 million inhabitants and 40,000 newborns estimated in 2019) to take care of these malformations. In the decade under investigation, the catchment population of our Department (spanning almost all Western Sicily, i.e. the provinces of Agrigento and Caltanissetta besides that of Palermo) was of about 175.000 newborns.

Data collected by neonatal records and follow-up charts were related to familiar and/or maternal risk factors (consanguinity, maternal preexisting and/or gestational diseases, exposure to teratogen/infectious agents, lack of preconception folic acid supplement), demographic (ethnicity/origin, residence), clinical features (eventual use of assisted reproduction techniques, prenatal diagnosis, gestational age, fetal presentation, type of delivery, birth weight, preoperative imaging, modalities of pharmacological prophylaxis and/or therapy with antibiotics and analgesics, description of the surgery intervention, length of hospital stay, comorbidities, complications), and neurodevelopmental outcomes. Ethical approval was granted from the Ethics Committee of our University Hospital.

## Results

Seven patients were included in this study (5 males and 2 females). All of them were admitted on the first day of life, 4 inborn, and 3 were transferred from other first level birth centers.Ethnicity was Sicilian for all newborns except for one, whose parents were from Ghana.

No consanguinity was found in any case. Five families lived in the urban area and two came from a rural area. All patients were naturally conceived. Two women with preexisting diseases were observed, one with severe obesity, and the other with HIV infection. No mother received preconception folic acid supplement, and only one woman took folate during pregnancy. A prenatal diagnosis was performed in only 2 (1 lumbosacral myelomeningocele, and 1 terminal myelocystocele) of 7 subjects. All newborns, except one (35 WG), were at term. All had cephalic presentation. Four were born by vaginal delivery (one of which was precipitous), and 3 by caesarean section. Two were low-birth-weight infants (< 2500 g). The length of hospitalization ranged from 8 to 22 days.

### Clinical features

**Fig. 1:**
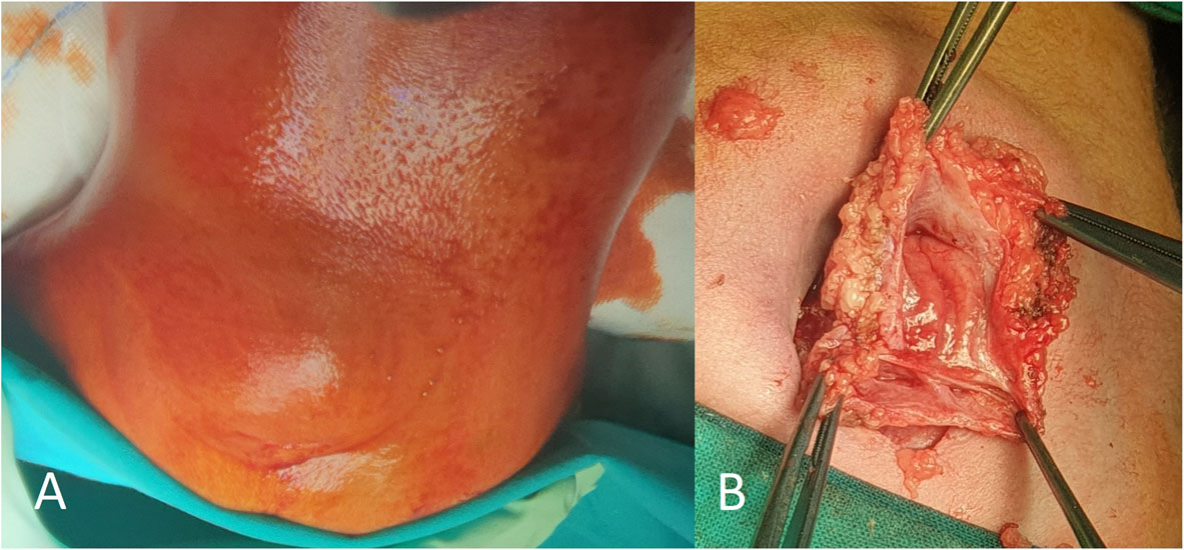
Female neonate with lumbosacral lipomyelomeningocele (**a**); an associated split cord malformation was evident during surgery (**b**)

A wide spectrum of NTDs was observed in our patients: three had open NTDs, namely myelomeningocele (MMC, 2 lumbosacral, one of which associated with extradural lipoma and concomitant split cord malformation, and 1 sacral), and 4 closed ones, including 2 with occipital meningocele, 1 filar lipoma associated with dermal sinus, and 1 terminal myelocystocele. Three patients had associated anomalies and/or comorbidities: 1 cerebellar and corpus callosum hypoplasia and feet deformities, with *normal* array comparative genomic hybridization (a-CGH) *findings,* 1 hypospadias, and 1 HIV infection, severe perinatal asphyxia, and congenital microcephaly (head circumference below 2 SD). Images related to the newborn with MMC and split cord malformation are showed in Fig. 1.

### Medical imaging

All patients performed in the first days of life ultrasound (US) examinations of brain, skin and soft tissues over the spine, abdomen and heart. The newborn affected by filar lipoma performed also lumbosacral X-ray. In the two patients with occipital meningocele, *Computed Tomography* (*CT*) of the *head in one case, and brain and spine Magnetic Resonance Imaging (MRI) in the other one, were performed* to rule out a cerebral parenchymal involvement*. Ophthalmological evaluation was performed in both of them. In the 2 newborns with MMC, cerebral US showed triventricular hydrocephalus along with cerebellar and corpus callosum hypoplasia in one subject, and isolated non progressive enlargement of the lateral ventricles in the other one. In the patient with myelocystocele an isolated cavitation of choroid plexus was observed.*

### Treatment and neurodevelopmental outcomes

A surgical procedure was performed in 5 of the 7 patients in neonatal age within 72 h of life, 3 with open NTDs and two with occipital meningocele. In three newborns with MMC, it consisted of a direct approach, preserving maximum healthy skin to allow a closure of the defect. An incision between the dura mater and the skin around the neural placode was performed. A progressive dissection and identification of the meningeal plan, until the basis of the bone cleft, was made. An opening of the dural sac was carried out, then the dura was sutured with very fine polydioxanone continuous suture before skin closure. One of these three patients underwent a contextual excision of a lipomatous extradural lesion in the upper third of the defect, another one a simultaneous ventriculoperitoneal shunting for concomitant hydrocephalus, and the third an additional lateral relaxing incisions in the fascia for primary skin defect closure. In the newborns with occipital meningocele, the intervention consisted of a simple section of the defect on its basis, with covering of the bone abnormality with surrounding tissue, and then closure of the scalp. All patients underwent parenteral antibiotic prophylaxis and analgesic therapy during surgical procedures and, except for the preterm one, who was further supported by mechanical ventilation, they were spontaneously breathing at the end of operating session.

The postoperative evolution was uneventful in all cases, with the exception of a newborn with MMC suffering from a surgical wound dehiscence, that healed in 10 days. No deaths occurred in the present case series. At follow-up, the two patients with MMC presented with the expected severe neurological impairment related to the spinal segment involvement above L3. A female patient aged 5 years, with concomitant cerebral anomalies, is also affected by a global developmental delay. The youngest patient with MMC, aged two months, has a reduced motility of lower limbs and fecal incontinence and no cerebral ventricular dilatation. The preterm patient with lumbosacral spina bifida, aged 5 years, achieved autonomic walking at two years of age, normal urinary and fecal continence, and shows a normal cognitive developmental level. The third newborn with MMC and associated extradural lipoma, does not have a significant follow-up time (as she currently is 3 months old), and actually shows hypoesthesia and reduced motility of the lower limbs. The male patient with isolated occipital meningocele underwent surgical correction at three months of age, and now, aged 7 years, has a normal neurodevelopmental profile. The male infant with filar lipoma and dermal sinus at three months of age shows a normal lower limbs motility and fecal continence. He has not yet been operated, in relation to the parental will to wait for a greater age. The patient with terminal myelocystocele was operated at three months of age, and now, aged 10 years, he has a normal global neurodevelopmental profile. Epidemiological and clinical data are summarized in Tables 1 and 2.

**Table 1:** Epidemiological data of present patients

SEX	• 5 males • 2 females
TYPE OF ADMISSION	• 4 inborn • 3 outborn
ORIGIN (ethnicity)	• 6 Sicilian (European) • 1 Ghanaian (African)
RESIDENCE	• 5 urban area • 2 rural area
PREEXISTING MATERNAL DISEASES	• 1 severe obesity • 1 HIV infection
FOLIC ACID SUPPLEMENT	• 0 preconception • 1 gestational

**Table 2:** Clinical characteristics of present patients

PRENATAL DIAGNOSIS	2/7 newborns • 1 lumbosacral myelomeningocele • 1 terminal myelocystocele
GESTATIONAL AGE	• 6 at term • 1 preterm (35 WG)
TYPE OF DELIVERY	• 4 vaginal (1 precipitous) • 3 cesarean section
TYPE OF NEURAL TUBE DEFECT	• 3 open: MMC, 2 lumbosacral, one of which associated with extradural lipoma, and 1 sacral • 4 closed: 2 occipital meningocele, 1 filar lipoma associated with dermal sinus, 1 terminal myelocystocele
ASSOCIATED ANOMALIES AND/OR COMORBIDITIES	3/7 newborns • 1 cerebellar and corpus callosum hypoplasia and feet deformities, with *normal* a-CGH findings • 1 hypospadias • 1 HIV infection, severe perinatal asphyxia, and congenital microcephaly (< −2SD)
MEDICAL IMAGING/SPECIALISTIC EVALUATIONS	• 7/7 US of brain, skin and soft tissues over the spine, abdomen and heart • 1/7 lumbosacral X-ray • *1/7 CT* of the *head* • *1/7 brain and spine MRI* • *2/7 ophthalmological evaluation*
SURGICAL PROCEDURE	5/7 newborns • 3 MMC • 2 meningocele
POSTOPERATIVE EVOLUTION	• 4/5 uneventful • 1/5 surgical wound dehiscence
NEURODEVELOPMENTAL OUTCOMES	• 3 normal neurodevelopmental profile and/or normal lower limbs motility and fecal continence • 2 severe neurological impairment • 1 global developmental delay • 1 reduced motility of lower limbs and fecal incontinence

## Discussion

The term “spina bifida” was first coined in 1641 by Nicholas Tulp [12]. The spread of surgical treatment of open NTDs and the knowledge of pathophysiology (including primary prevention through folic acid preconception supplement), epidemiology and genetics of such defects were reached much later, in the late twentieth century [9].

Most NTDs are sporadic and multifactorial: an oligogenic/polygenic inheritance (genes regulating folate and methionine metabolism, and others involved in planar cell polarity signaling pathway) interact with influencing environmental factors [4, 5, 13]. Risk factors include: maternal folate deficiency, diabetes, obesity, exposure to teratogens (valproic acid and carbamazepine, lead and tetrachloroethylene-contaminated drinking water, arsenic, pesticides, mycotoxins, fungus contaminants of maize, heat, influenza virus), specific parental occupations and low socioeconomic status [4, 14]. Recurrence risk of a second affected child is increased by 3–5 folds for couples with one sick infant, compared to the general population. A significant proportion (10%) of NTDs are associated with syndromes and/or chromosomal anomalies/congenital malformations, and the correlation is more marked (20%) in some ethnic groups (i.e. Arab populations with increased prevalence of autosomal recessive diseases, reflecting their high coefficient of consanguinity) [15]. Associated conditions included are: VACTERL association (vertebral abnormalities, anal atresia, cardiac defects, tracheal anomalies including tracheo-esophageal fistula, esophageal atresia, renal anomalies, limb anomalies, MIM:192350), amniotic band sequence, Currarino syndrome (a peculiar form of caudal regression syndrome, also known as autosomal dominant sacral agenesis, MIM:176450), Waardenburg syndrome, Joubert syndrome, Meckel-Gruber syndrome and some chromosomal abnormalities like trisomy 13, trisomy 18, triploidy and partial aneuploidy [16].

The low number of patients affected/families involved limited the diagnostic efficacy of genetic analysis. However, whole genome sequencing techniques found candidate NTDs *loci* and genes in chromosomes 2, 7, 10 and 17 (some of which, i.e. *WIPI1*, *SPHKAP* and *NCOR1*, have recently been associated with anencephaly) [7, 16]. These insights add to our existing knowledge of the genetic mechanisms underlying NTDs development [17]. The ongoing genomic revolution will indeed offer the possibility to carry out genomic projects, with the aim of identifying new variants implicated in the biologic basis of NTDs [16–18].

The low rate of NTDs observed in our case series, even more marked than that currently found in HICs [8, 19–21], may be explained by some plausible factors: a likely high number of prenatal diagnoses and abortions in our setting, and the phenomenon of health emigration (from Sicily to central-northern Italian regions), that may have contributed to the reduction/underestimation of our cases.

A large number of studies provided evidence that folate fortification reduces the incidence of MMC and anencephaly (conditions known as folic acid preventable spina bifida, FAP-SB, which represent up to 92% of all NTDs) [9, 22–24]. In USA preventive policies were first implemented in 1992, with a recommendation by the Public Health Service, that all women of childbearing age should consume 400 μg of folic acid daily [9]. In 1998, mandatory fortification of enriched cereal grain products was instituted, whose adoption resulted in a 28% reduction in NTDs prevalence (from 10.7 cases/1000 in 1992 to 7/1000 in 1998) [9]. However, after the initial reduction, birth prevalence remained stable since 1999, with nearly a quarter (21.6%) of childbearing age women who still have low red blood folate concentration associated with a higher risk for NTDs [9]. Furthermore, fortification mandates are still not universally adopted, and most countries (mainly LMICs) are not fortifying grains or rice with folate. Consequently, the rate of actual prevention of FAP-SB is only 13% [9, 24]. Therefore, also in presence of a low number of cases of NTDs, like those found in our report, prevention measures need to be improved and implemented even in HICs, also in light of the increased risks of short and long-term morbidity and disability affecting these subjects. These unfavorable outcomes are still highly prevalent also in HICs [9, 19]. Thus, despite the achievement of advanced standards of care and the improvements in neonatological management and surgical techniques [8, 25, 26], they have been observed also in some of our patients.

Newborns and children with NTDs need a multidisciplinary approach and follow-up, involving neonatologists, neonatal surgeons, pediatric neurologists, urologists and orthopedics, geneticists, physiatrists, physiotherapists and neurorehabilitation therapists. Our multidisciplinary team aims to guarantee to children and families, an individualized care oriented to maintain normal biologic functions and social life by preserving urinary and stool continence, lowering the possible infective risk, and planning and a neurodevelopmental follow-up.

## Conclusions

The awareness of the benefits of preconception folic acid supplement is still not optimum even in HICs [23]. This is consistent with the data of our report, which showed a low rate/absence of folate supplement among the mothers of our patients. Adequate medical counselling may not reach women with unplanned pregnancies, and/or more vulnerable owing to socio-economic status. Since even in HICs, prenatal US screening cannot completely contain the impact on health systems that these defects exert in relation to the burden of complex care and morbidity/disability risks. These primary intervention remains our most important and effective tool. Thus, alternative preventive strategies should be considered. These may include fortifying commonly consumed foods and, through public health policies, allow a free availability of preconception folic acid supplements to all women of childbearing age.

Neonatologists and pediatricians should claim a leading role in national and international health as well as research policies, with the goal of preventive and curative care pertaining to disability.

Every child has the right to be protected against any disease, even more if complex and burdened by high morbidity and mortality. This is particularly true for newborns with NTDs, for whom we have an extraordinary potential of prevention available. If this is not achieved, an early, multidisciplinary, individualized and family centered as well as longitudinal management, should be guaranteed.

## Abbreviations

a-CGH: Array comparative genomic hybridization
CT: Computed tomography
FAP-SB: Folic acid preventable spina bifida
HICs: High income countries
LMICs: Low and middle income countries
MMC: Myelomeningocele
MRI: Magnetic resonance imaging
NICU: Neonatal intensive care unit
NTDs: Neural tube defects
US: Ultrasound
USA: United States of America
WG: Weeks of gestation
